# Supplementary material for: Human NOP2/NSUN1 regulates ribosome biogenesis through non-catalytic complex formation with box C/D snoRNPs
Source: Nucleic Acids Res. 2022 Sep 26;50(18):10695–716. doi: 10.1093/nar/gkac817 (PMC9561284; doi:10.1093/nar/gkac817)
Supplement: gkac817_Supplemental_Files [file gkac817_supplemental_files.zip › Liao_et_al_Supplementary_Figure_Legend_clean.docx]

**Supplementary Figure S1.** NOP2/NSUN1 binds to C/D box snoRNAs. **(A)** HEK293T cells expressing FLAG-tagged NOP2/NSUN1 WT or C459A mutant were fixed and immunostained with an anti-FLAG (green) and anti-FBL (red, nucleolar marker). DNA was visualized by staining with Hoechst 33342 (blue). The scale bar is representative of all panels: 10 μm. **(B)** HEK293T cells expressing FLAG-tagged NOP2/NSUN1 WT or C459A mutant were lysed and immunoprecipitated (IP) with an anti-FLAG antibody. Associated RNAs were analyzed by RT-qPCR with specific primers as indicated. Mock transfected cells immunoprecipitated (IP) with an anti-FLAG antibody were used as negative control. Enrichment was calculated over IgG control. Data are presented as the mean of 3 independent biological replicates ± standard deviation (SD). Statistical significance between Mock and WT or C459A mutant IP samples was calculated using a 2-tailed independent student *t*-test.

**Supplementary Figure S2.** NOP2/NSUN1 does not crosslink to C/D box snoRNAs. miCLIP-sequencing RT stops and mapped reads on SNORD3/U3 and SNORD118/U8. miCLIP-sequencing data were aligned to human SNORD3/U3 (ENST00000620232) or SNORD118/U8 (ENST00000363593). The mapping information was retrieved using Samtools. Reverse transcription (RT) stops on SNORD3/U3 **(A)**, SNORD118/U8 **(B)** per 1000 mapped reads were plotted by assigning the start (+1) sites of their respective Read1 sequence. miCLIP reads mapped to SNORD3/U3 **(C)** and SNORD118/U8 **(D)** in NOP2/NSUN1 WT or C459A mutant IP samples normalized to the total mapped reads of each corresponding sample.

**Supplementary Figure S3.** NOP2/NSUN1 is required for pre-rRNA processing. **(A)** HCT116 cells were transfected with non-targeting control (siC), NOP2 siRNA #1 or #2. After 72h, total RNA was separated on formaldehyde denaturing agarose gel and analyzed by Northern blot with 5’ETS, 3’ETS, ITS-1, ITS-2, 18S, 28S, and 7SL probes. A fraction of cells was collected to assess NOP2/NSUN1 depletion efficiency by Western blot (WB). **(B)** Densitometry quantification of each rRNA precursor from (A) normalized to 7SL RNA. The data are presented as the mean of 3 independent biological replicates ± standard deviation (SD). Statistical significance between NOP2/NSUN1 depleted samples and non-targeting siRNA control samples was calculated using a 2-tailed independent student *t*-test.

**Supplementary Figure S4.** NOP2/NSUN1 depletion impairs pre-rRNA processing but does not affect SNORD3/U3 and SNORD118/U8 stability **(A)** Densitometry ratio between each two rRNA precursors from Northern blot in Figure S3A. Data are presented as the mean of 3 independent biological replicates ± standard deviation (SD). Statistical significance between NOP2/NSUN1 depleted samples and non-targeting control samples was calculated using a 2-tailed independent student *t*-test. **(B)** Different exposure of Northern blots from Figure 5B. **(C)** RNA samples from Figure S3A were separated on urea-PAGE gel and analyzed by Northern blot with SNORD118/U8, SNORD3/U3, 5S, 5.8S, and 7SL specific probes. **(D)** Densitometry quantification of SNORD118/U8, SNORD3/U3, 5S, and 5.8S signal from (B) normalized to 7SL RNA signal. Data are presented as the mean of 3 independent biological replicates ± standard deviation (SD). Statistical significance between NOP2/NSUN1 depleted samples and non-targeting control samples was calculated using a 2-tailed independent student *t*-test.

**Supplementary Figure S5.** NOP2/NSUN1 cysteine 513 is required to catalyze m5C modification at position 4447 of the 28S rRNA. **(A)** HCT116 cells expressing empty vector (Vector), siRNA resistant NOP2/NSUN1 WT, or C513A mutant were transfected with non-targeting control (siC) or NOP2 siRNA #2. After 72h, total RNA was extracted and NOP2/NSUN1 depletion was assessed by RT-qPCR. Primers specific to the *NOP2* coding sequence (CDS) were used to detect both endogenous and exogenous *NOP2* and primers specific to *NOP2* 3’UTR were used to detect endogenous *NOP2* only. Expression of *ACTB* was used for normalization. RT-qPCR data are presented as the mean of 3 independent biological replicates ± standard deviation (SD). Statistical significance between NOP2 depleted samples and non-targeting siRNA control samples was calculated using a 2-tailed independent student t-test. **(B)** A fraction of cells from (A) were used to determine NOP2/NSUN1 depletion efficiency by Western blot with the indicated antibodies. **(C)** A fraction of cells from (A) were harvested for nuclear RNA extraction. Nuclear RNA was treated with bisulfite salt and sequenced on the Illumina Mi-Seq platform. Numbers of non-converted (5mC modification) and converted cytosines (unmethylated C) for each cytosine position near C4447 were plotted. Yellow marks highlight converted cytosines at position 4447. **(D)** Percentage of converted cytosine (C) at position 4447 on the 28S rRNA in each sample. Data are shown as the mean of 2 independent biological replicates ± standard deviation (SD). Statistical significance values relative to empty vector + NOP2/NSUN1 knockdown (Vector siNOP2#2) were calculated using a 2-tailed independent student t-test.

**Supplementary Figure S6.** Depletion of NOP2/NSUN1 by inducible shRNA causes rRNA processing defects. **(A)** HCT116 cells expressing doxycycline-inducible NOP2 shRNA were induced with 200 ng/ml doxycycline (Dox+) for 4 days. Non-induced (Dox-) cells were used as control. Total RNA was extracted and separated on formaldehyde denaturing agarose gel and analyzed by Northern blot with 5’ETS, 3’ETS, ITS-1, ITS-2, 18S, 28S, and 7SL probes. A fraction of cells was collected to determine NOP2/NSUN1 depletion efficiency by Western blot (WB) with the indicated antibodies. **(B)** Densitometry quantification of each rRNA precursor from (A) normalized to 7SL RNA. The data are presented as the mean of 3 independent biological replicates ± standard deviation (SD). Statistical significance between NOP2 depleted samples (Dox+) and non-induced control samples (Dox-) were calculated using a 2-tailed independent student t-test.

**Supplementary Figure S7.** NOP2/NSUN1 is required to maintain the integrity of C/D box snoRNPs. HCT116 cells were transfected with non-targeting control (siC), NOP2 siRNA #1 or #2. 72h later, cells were lysed and immunoprecipitated (IP) with anti-NOP56 antibody **(A, B)** or 15.5K antibody **(C, D)**. A fraction of the immuno-precipitate was analyzed by Western blot to control for immunoprecipitation and knockdown efficiency **(A, C)**. The remaining immunoprecipitated fraction was processed for RNA extraction. NOP56 **(B)** or 15.5K **(D)** associated RNA was analyzed by RT-qPCR with SNORD3/U3, SNORD118/U8, SNORD12, SNORD14C, and SNORD28 specific primers. Relative enrichment over IgG control is represented. The Data is presented as the mean of 3 independent biological replicates ± standard deviation (SD). Statistical significance between NOP2/NSUN1 depleted samples and non-targeting siRNA control samples was calculated using a 2-tailed independent student *t*-test. **(E)** Densitometry quantification of each protein in input samples from (A) normalized to GAPDH. Data are presented as the mean of 3 independent biological replicates ± standard deviation (SD). Statistical significance between NOP2 depleted samples and non-targeting control samples was calculated using a 2-tailed independent student t-test.

**Supplementary Figure S8.** Human NOP2/NSUN1 is predominantly located in nucleoli. **(A)** Proteins in nuclear extracts, nucleolar extracts, and insoluble nucleoli pellets from HCT116 cells were analyzed by Western blot with the indicated antibodies. **(B)** HCT116 cells were fixed and immunostained with anti-NOP2/NSUN1 (green) and anti-FBL (red, nucleolar marker). DNA was visualized by staining with Hoechst 33342 (blue). Scale bar is representative of all panels: 10 μm.

**Supplementary Figure S9.** Northern blot analysis of rRNA precursors present in each fraction from the sucrose gradient fractionation from Figure 9A. RNA from fractions 1-12 was extracted, separated on formaldehyde denaturing agarose gel, and analyzed by Northern blot with ITS1 **(A)** and ITS2 **(B)** probes.

**Supplementary Figure S10.** Pre-rRNA processing defects observed after NOP2/NSUN1 depletion are independent of p53 status. HCT116 p53 -/- cells were transfected with non-targeting control (siC), NOP2 siRNA #1 or #2. 72h later, total RNA was separated on formaldehyde denaturing agarose gel and analyzed by Northern blot with 5’ETS, ITS-1, and ITS-2 probes. Right panel: 28S and 18S rRNA were stained with methylene blue on the membrane.

**Supplementary Table 1.** Sequence of primers and probes used in this paper.

**Supplementary Table 2.** miCLIP sequencing Peaks with IP vs SMInput fold-change >= 2 and adjusted P value < 0.05.
