# Supplementary figures and images for: Human NOP2/NSUN1 regulates ribosome biogenesis through non-catalytic complex formation with box C/D snoRNPs
Source: Nucleic Acids Res. 2022 Sep 26;50(18):10695–716. doi: 10.1093/nar/gkac817 (PMC9561284; doi:10.1093/nar/gkac817)

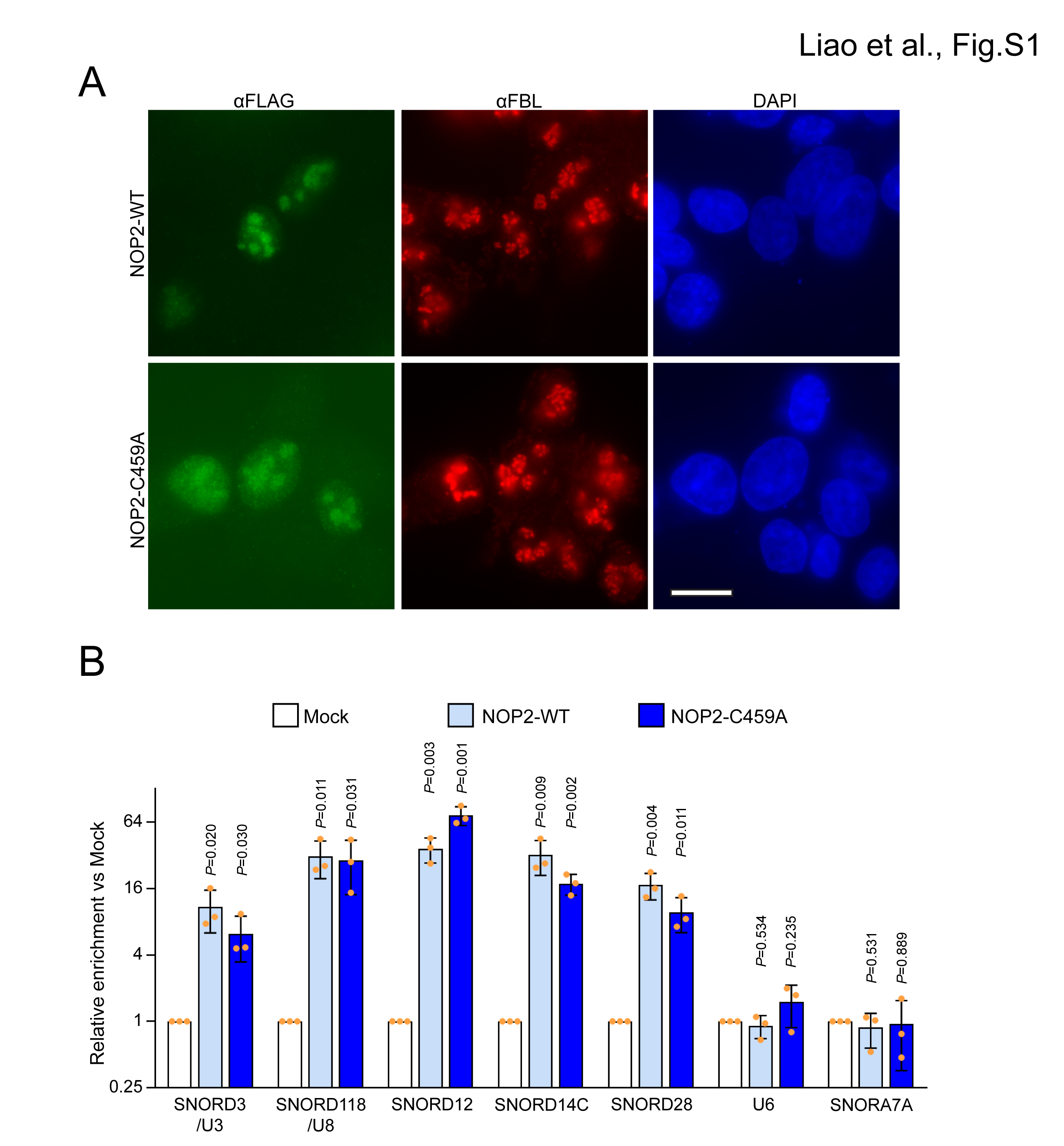

Supplement: gkac817_Supplemental_Files [file gkac817_supplemental_files.zip › Nop2FigS1-20220803-01.tiff]

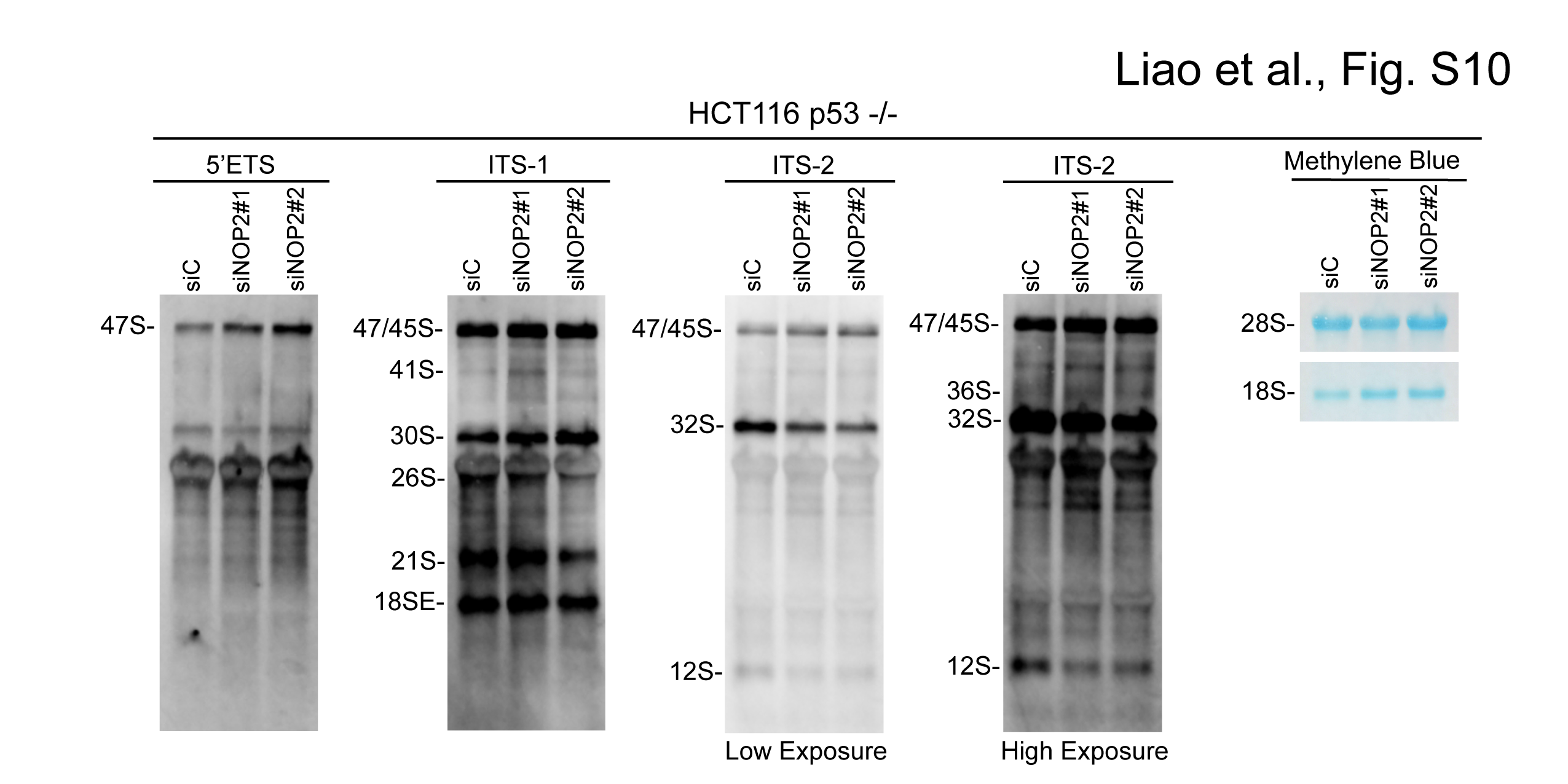

Supplement: gkac817_Supplemental_Files [file gkac817_supplemental_files.zip › Nop2FigS10-20220803-01.tiff]

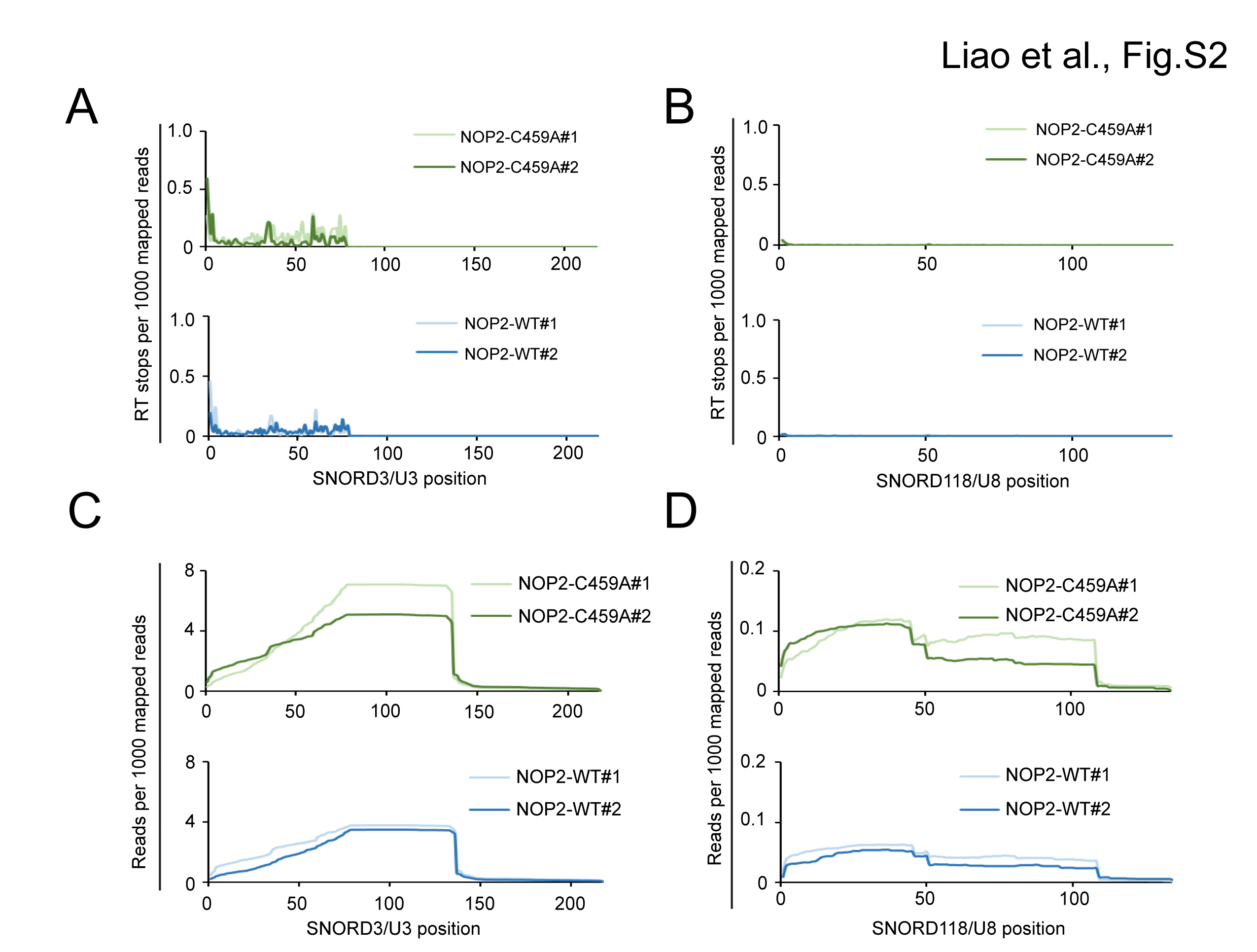

Supplement: gkac817_Supplemental_Files [file gkac817_supplemental_files.zip › Nop2FigS2-20220803-01.tiff]

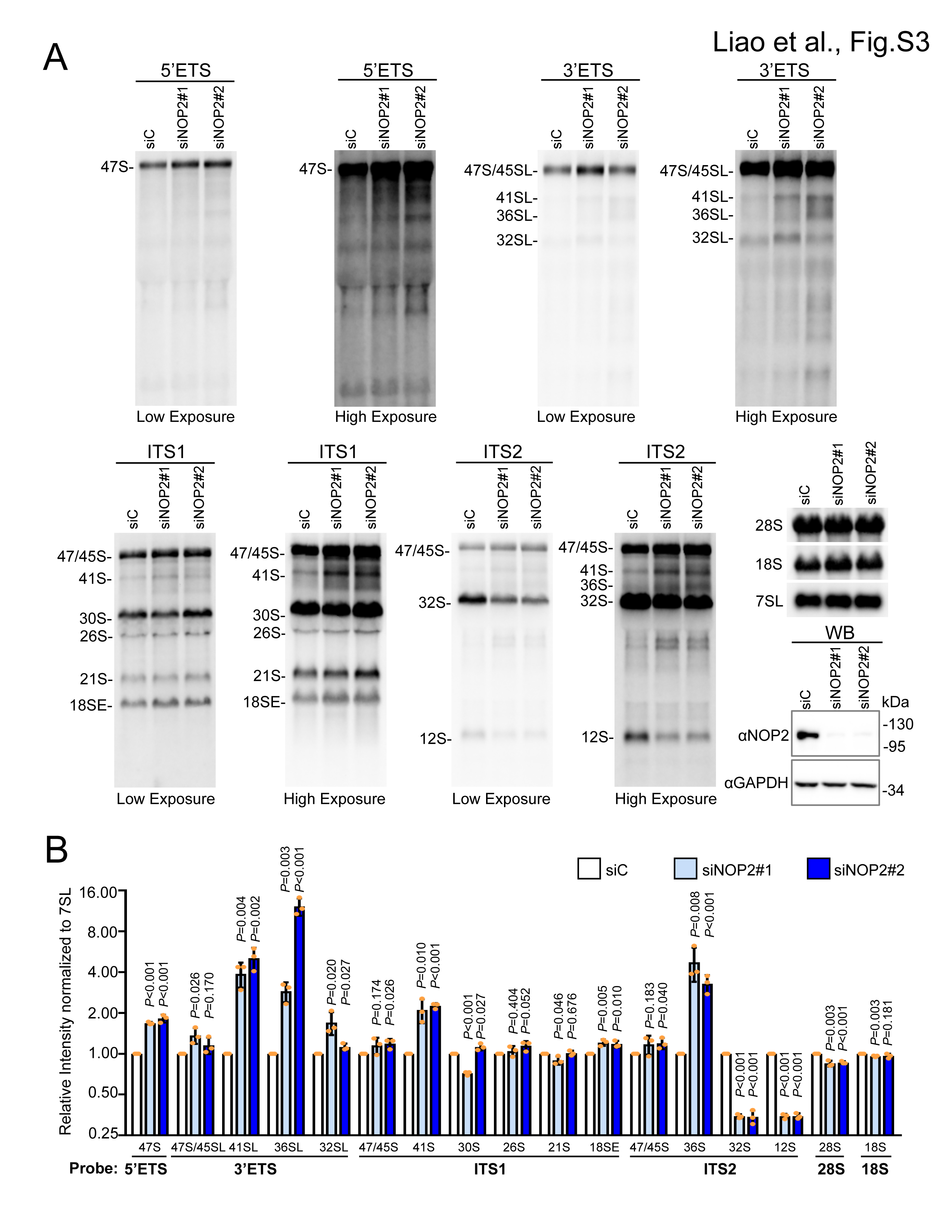

Supplement: gkac817_Supplemental_Files [file gkac817_supplemental_files.zip › Nop2FigS3-20220707-01.tiff]

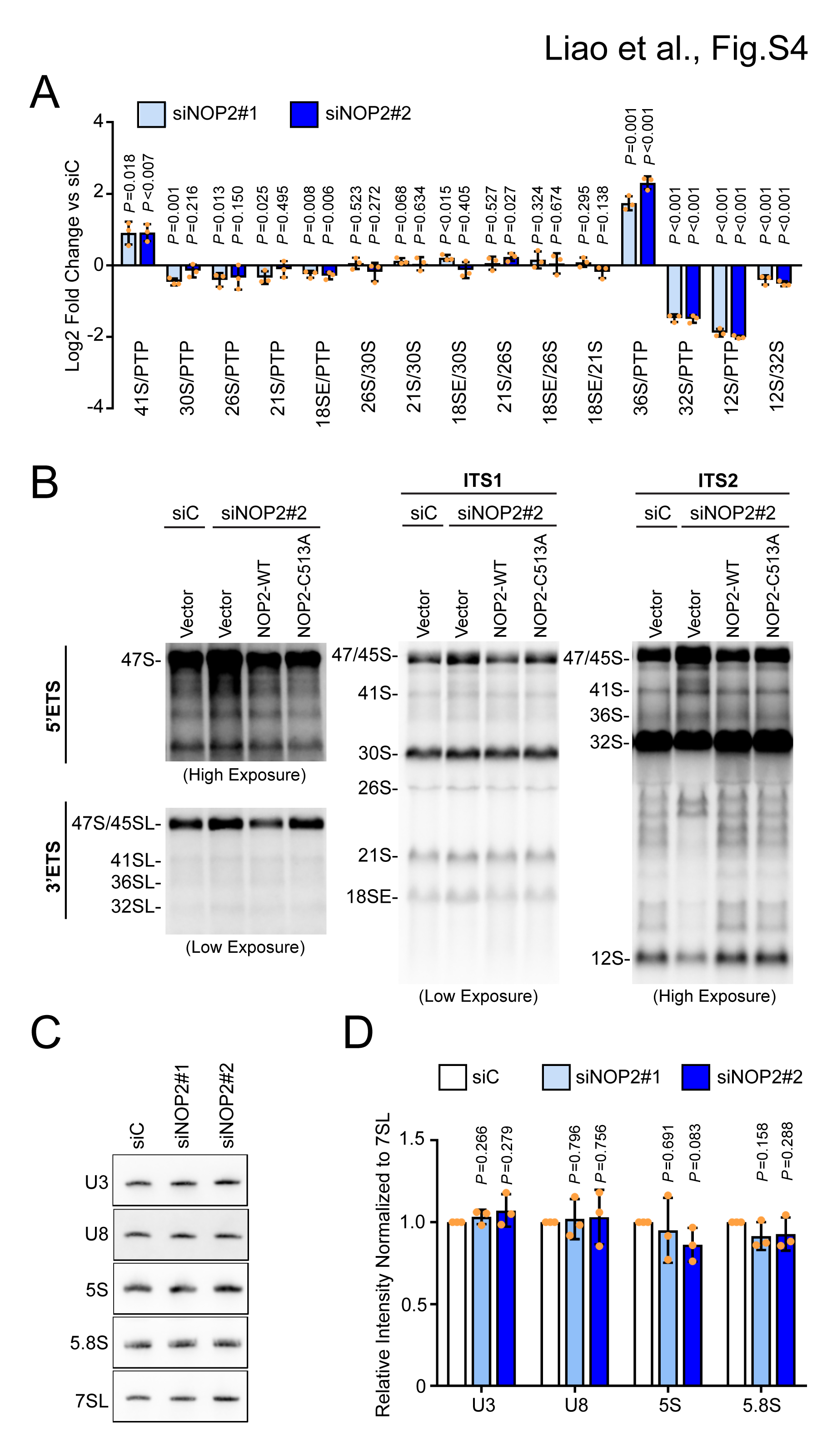

Supplement: gkac817_Supplemental_Files [file gkac817_supplemental_files.zip › Nop2FigS4-20220803-01.tiff]

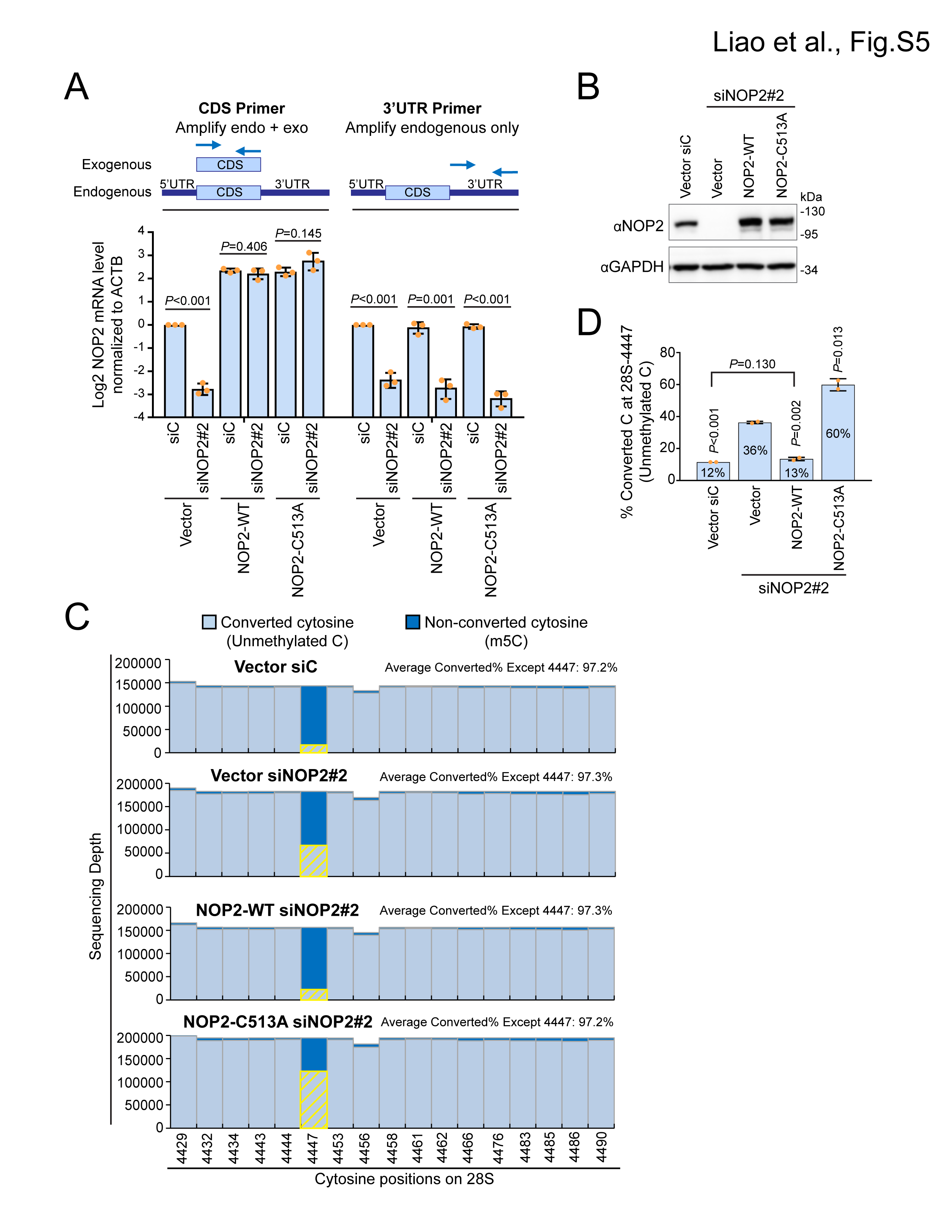

Supplement: gkac817_Supplemental_Files [file gkac817_supplemental_files.zip › Nop2FigS5-20220803-01.tiff]

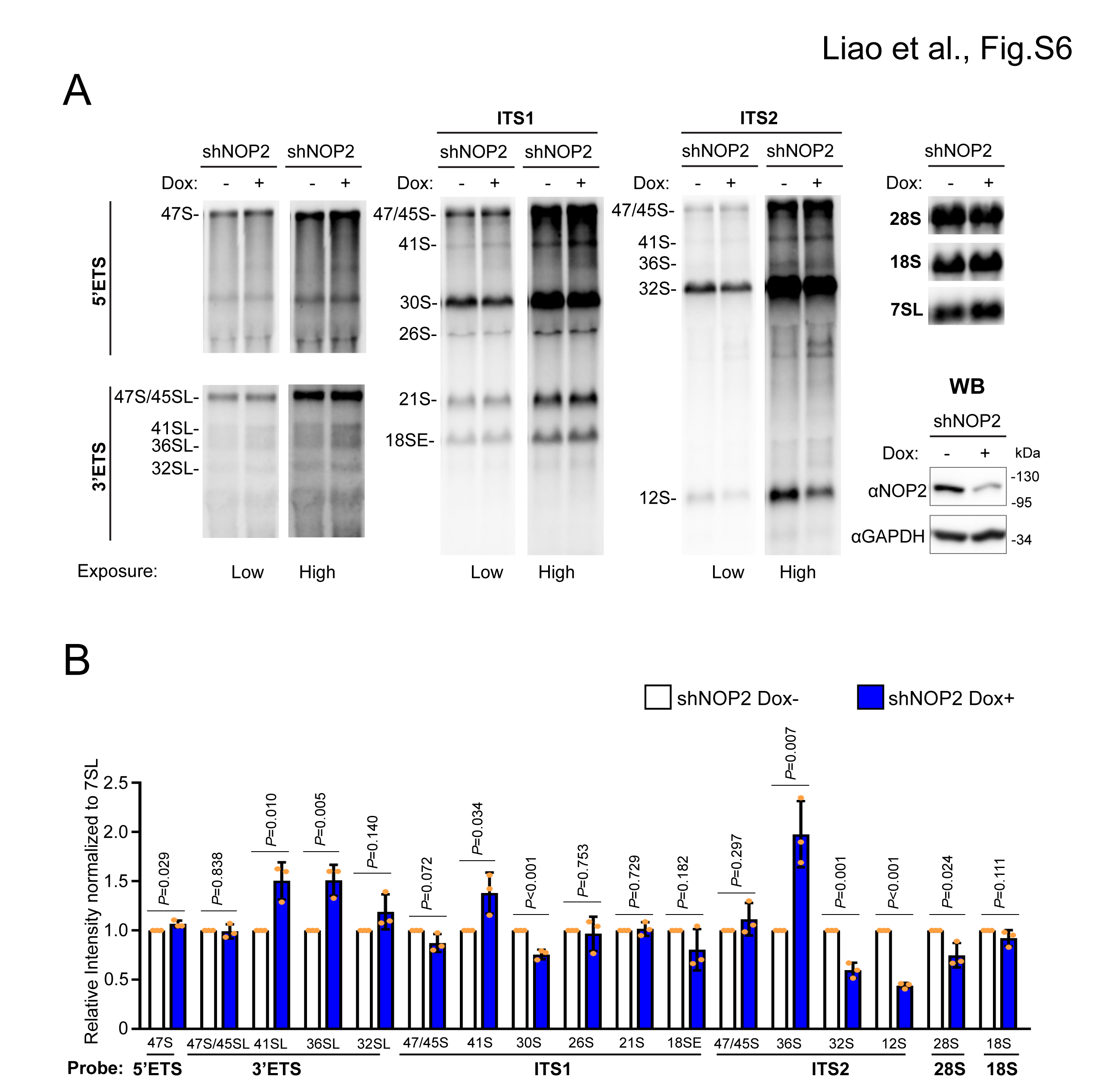

Supplement: gkac817_Supplemental_Files [file gkac817_supplemental_files.zip › Nop2FigS6-20220803-01.tiff]

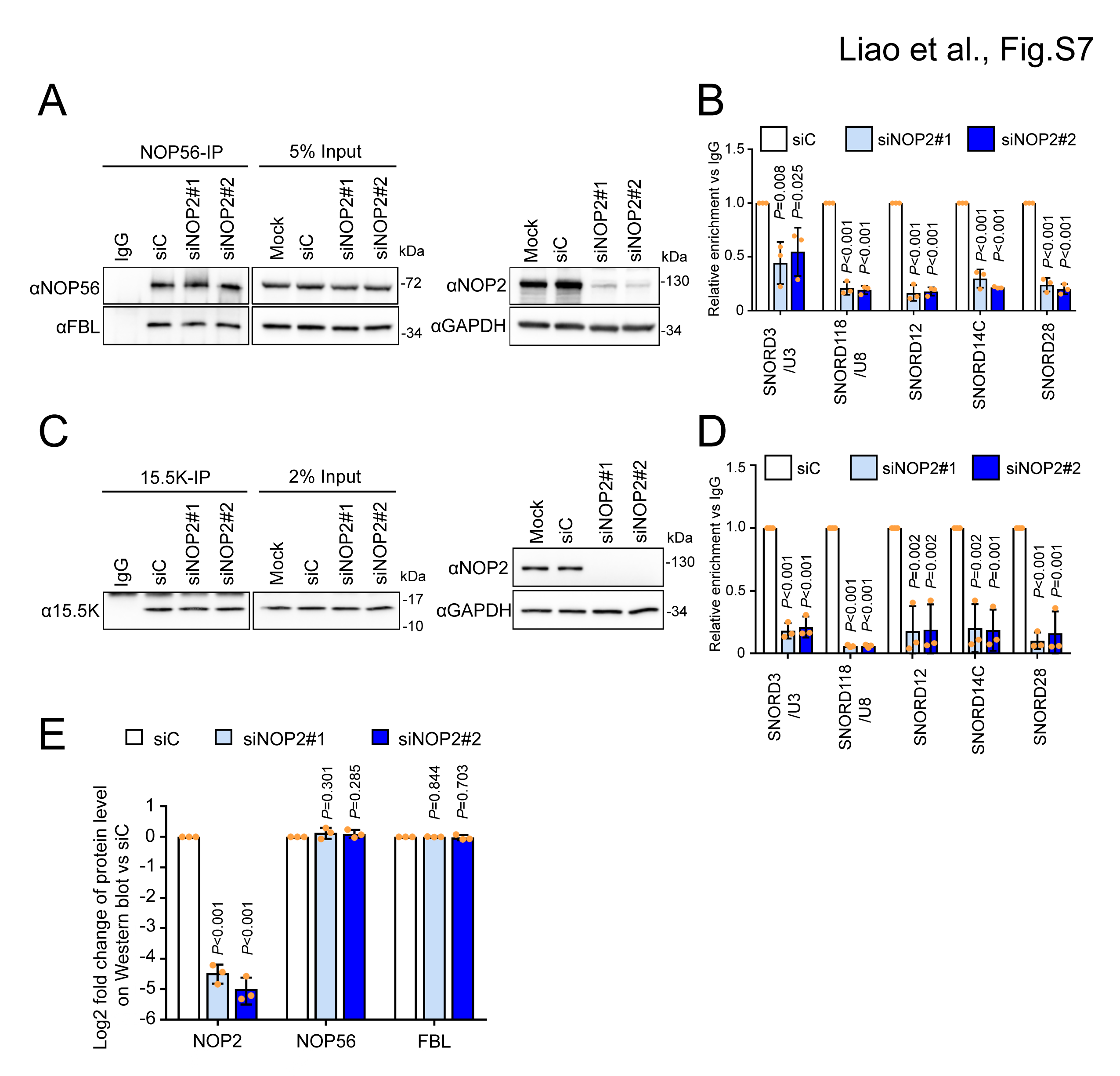

Supplement: gkac817_Supplemental_Files [file gkac817_supplemental_files.zip › Nop2FigS7-20220707-01.tiff]

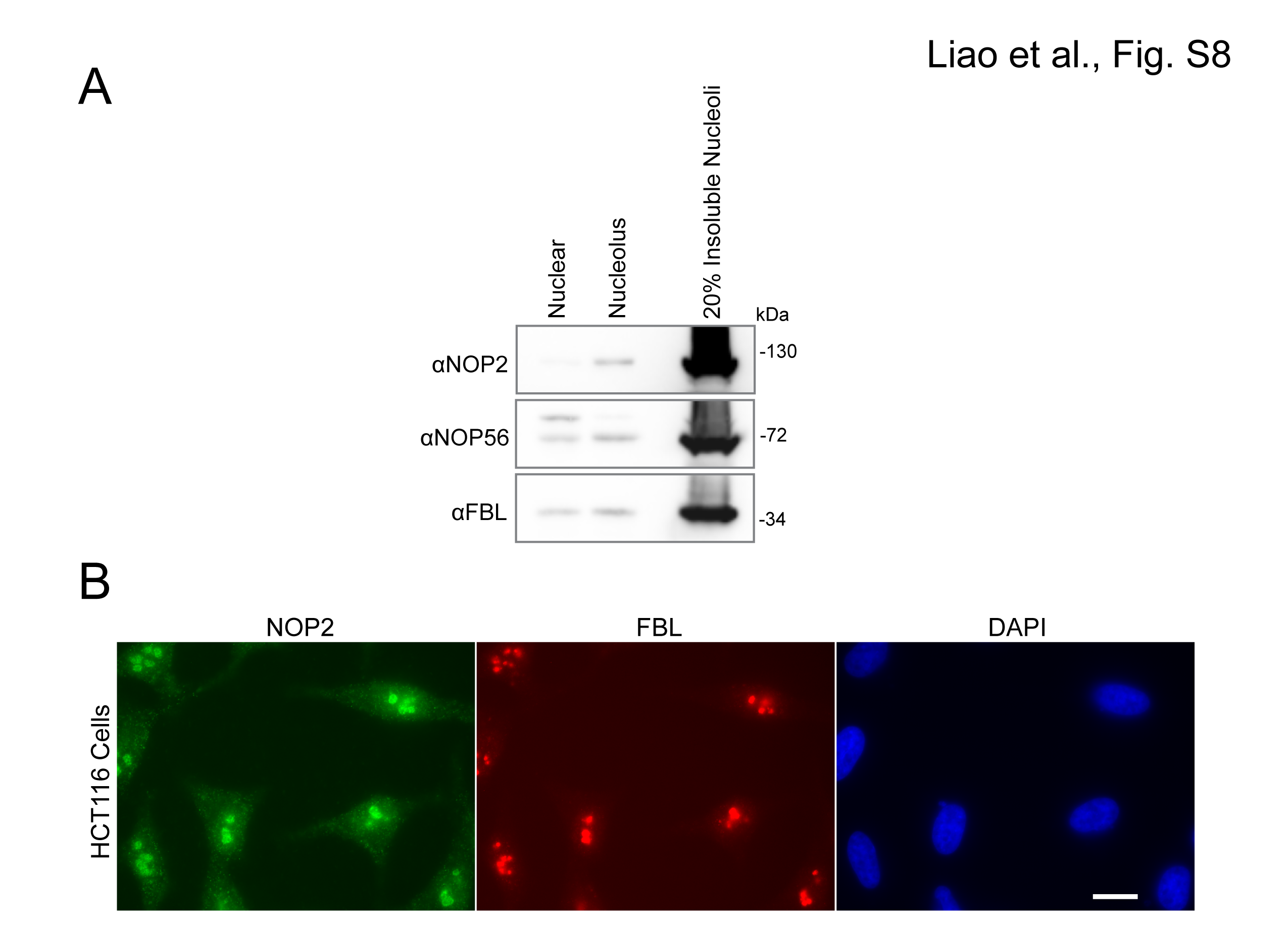

Supplement: gkac817_Supplemental_Files [file gkac817_supplemental_files.zip › Nop2FigS8-20220801-01.tiff]

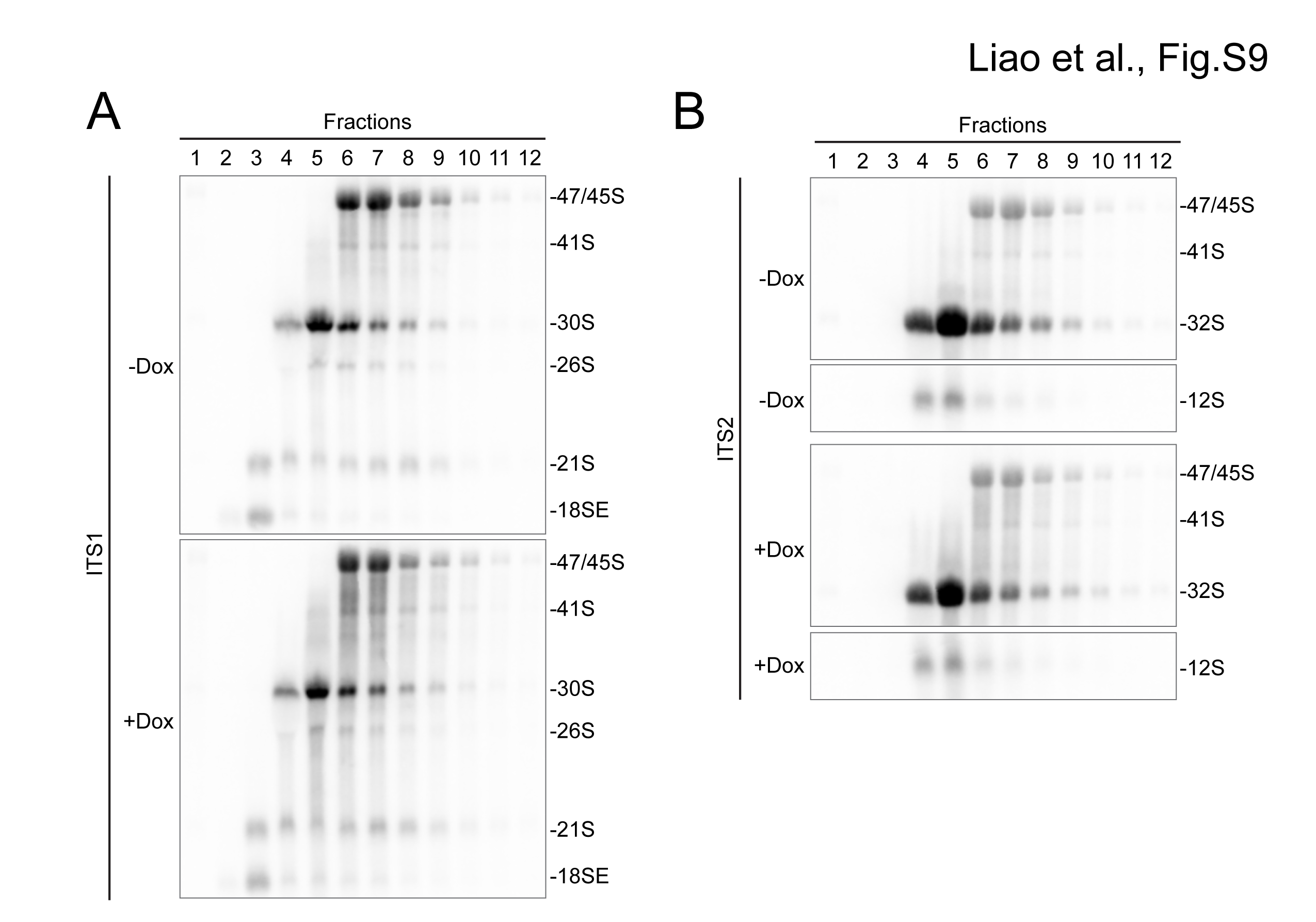

Supplement: gkac817_Supplemental_Files [file gkac817_supplemental_files.zip › Nop2FigS9-20220803-01.tiff]
